# Equilibrium Propagation: Bridging the Gap between Energy-Based Models and Backpropagation

**DOI:** 10.3389/fncom.2017.00024

**Published:** 2017-05-04

**Authors:** Benjamin Scellier, Yoshua Bengio

**Affiliations:** Département d'Informatique et de Recherche Opérationnelle, Montreal Institute for Learning Algorithms, Université de MontréalMontreal, QC, Canada

**Keywords:** artificial neural network, backpropagation algorithm, biologically plausible learning rule, contrastive hebbian learning, deep learning, fixed point, Hopfield networks, spike-timing dependent plasticity

## Abstract

We introduce Equilibrium Propagation, a learning framework for energy-based models. It involves only one kind of neural computation, performed in both the first phase (when the prediction is made) and the second phase of training (after the target or prediction error is revealed). Although this algorithm computes the gradient of an objective function just like Backpropagation, it does not need a special computation or circuit for the second phase, where errors are implicitly propagated. Equilibrium Propagation shares similarities with Contrastive Hebbian Learning and Contrastive Divergence while solving the theoretical issues of both algorithms: our algorithm computes the gradient of a well-defined objective function. Because the objective function is defined in terms of local perturbations, the second phase of Equilibrium Propagation corresponds to only nudging the prediction (fixed point or stationary distribution) toward a configuration that reduces prediction error. In the case of a recurrent multi-layer supervised network, the output units are slightly nudged toward their target in the second phase, and the perturbation introduced at the output layer propagates backward in the hidden layers. We show that the signal “back-propagated” during this second phase corresponds to the propagation of error derivatives and encodes the gradient of the objective function, when the synaptic update corresponds to a standard form of spike-timing dependent plasticity. This work makes it more plausible that a mechanism similar to Backpropagation could be implemented by brains, since leaky integrator neural computation performs both inference and error back-propagation in our model. The only local difference between the two phases is whether synaptic changes are allowed or not. We also show experimentally that multi-layer recurrently connected networks with 1, 2, and 3 hidden layers can be trained by Equilibrium Propagation on the permutation-invariant MNIST task.

## 1. Introduction

The Backpropagation algorithm to train neural networks is considered to be biologically implausible. Among other reasons, one major reason is that Backpropagation requires a special computational circuit and a special kind of computation in the second phase of training. Here, we introduce a new learning framework called Equilibrium Propagation, which requires only one computational circuit and one type of computation for both phases of training. Just like Backpropagation applies to any differentiable computational graph (and not just a regular multi-layer neural network), Equilibrium Propagation applies to a whole class of energy based models (the prototype of which is the continuous Hopfield model).

In Section 2, we revisit the continuous Hopfield model (Hopfield, 1984) and introduce Equilibrium Propagation as a new framework to train it. The model is driven by an energy function whose minima correspond to preferred states of the model. At prediction time, inputs are clamped and the network relaxes to a fixed point, corresponding to a local minimum of the energy function. The prediction is then read out on the output units. This corresponds to the first phase of the algorithm. In the second phase of the training framework, when the target values for output units are observed, the outputs are nudged toward their targets and the network relaxes to a new but nearby fixed point which corresponds to slightly smaller prediction error. The learning rule, which is proved to perform gradient descent on the squared error, is a kind of contrastive Hebbian learning rule in which we *learn* (make more probable) the second-phase fixed point by reducing its energy and *unlearn* (make less probable) the first-phase fixed point by increasing its energy. However, our learning rule is not the usual contrastive Hebbian learning rule and it also differs from Boltzmann machine learning rules, as discussed in Sections 4.1 and 4.2.

During the second phase, the perturbation caused at the outputs propagates across hidden layers in the network. Because the propagation goes from outputs backward in the network, it is better thought of as a “back-propagation.” It is shown by Bengio and Fischer (2015) and Bengio et al. (2017) that the early change of neural activities in the second phase corresponds to the propagation of error derivatives with respect to neural activities. Our contribution in this paper is to go beyond the early change of neural activities and to show that the second phase also implements the (back)-propagation of error derivatives with respect to the synaptic weights, and that this update corresponds to a form of spike-timing dependent plasticity, using the results of Bengio et al. (2017).

In Section 3, we present the general formulation of Equilibrium Propagation: a new machine learning framework for energy-based models. This framework applies to a whole class of energy based models, which is not limited to the continuous Hopfield model but encompasses arbitrary dynamics whose fixed points (or stationary distributions) correspond to minima of an energy function.

In Section 4, we compare our algorithm to the existing learning algorithms for energy-based models. The recurrent back-propagation algorithm introduced by Pineda (1987) and Almeida (1987) optimizes the same objective function as ours but it involves a different kind of neural computation in the second phase of training, which is not satisfying from a biological perspective. The contrastive Hebbian learning rule for continuous Hopfield nets described by Movellan (1990) suffers from theoretical problems: learning may deteriorate when the free phase and clamped phase land in different modes of the energy function. The Contrastive Divergence algorithm (Hinton, 2002) has theoretical issues too: the CD_1_ update rule may cycle indefinitely (Sutskever and Tieleman, 2010). The equivalence of back-propagation and contrastive Hebbian learning was shown by Xie and Seung (2003) but at the cost of extra assumptions: their model requires infinitesimal feedback weights and exponentially growing learning rates for remote layers.

Equilibrium Propagation solves all these theoretical issues at once. Our algorithm computes the gradient of a sound objective function that corresponds to local perturbations. It can be realized with leaky integrator neural computation which performs both *inference* (in the first phase) and *back-propagation of error derivatives* (in the second phase). Furthermore, we do not need extra hypotheses such as those required by Xie and Seung (2003). Note that algorithms related to ours based on infinitesimal perturbations at the outputs were also proposed by O'Reilly (1996) and Hertz et al. (1997).

Finally, we show experimentally in Section 5 that our model is trainable. We train recurrent neural networks with 1, 2, and 3 hidden layers on MNIST and we achieve 0.00% training error. The generalization error lies between 2 and 3% depending on the architecture. The code for the model is available^1^ for replicating and extending the experiments.

## 2. The continuous hopfield model revisited: equilibrium propagation as a more biologically plausible backpropagation

In this section, we revisit the continuous Hopfield model (Hopfield, 1984) and introduce Equilibrium Propagation, a novel learning algorithm to train it. Equilibrium Propagation is similar in spirit to Backpropagation in the sense that it involves the propagation of a signal from output units to input units backward in the network, during the second phase of training. Unlike Backpropagation, Equilibrium Propagation requires only one kind of neural computations for both phases of training, making it more biologically plausible than Backpropagation. However, several points still need to be elucidated from a biological perspective. Perhaps the most important of them is that the model described in this section requires symmetric weights, a question discussed at the end of this paper.

### 2.1. A kind of hopfield energy

Previous work (Hinton and Sejnowski, 1986; Friston and Stephan, 2007; Berkes et al., 2011) has hypothesized that, given a state of sensory information, neurons are collectively performing inference: they are moving toward configurations that better “explain” the observed sensory data. We can think of the neurons' configuration as an “explanation” (or “interpretation”) for the observed sensory data. In the energy-based model presented here, that means that the units of the network gradually move toward lower energy configurations that are more probable, given the sensory input and according to the current “model of the world” associated with the parameters of the model.

We denote by *u* the set of units of the network^2^, and by θ = (*W, b*) the set of free parameters to be learned, which includes the synaptic weights *W*_*ij*_ and the neuron biases *b*_*i*_. The units are continuous-valued and would correspond to averaged voltage potential across time, spikes, and possibly neurons in the same minicolumn. Finally, ρ is a non-linear activation function such that ρ(*u*_*i*_) represents the firing rate of unit *i*.

We consider the following energy function *E*, a kind of Hopfield energy, first studied by Bengio and Fischer (2015), Bengio et al. (2015a,b), and Bengio et al. (2017):

(1) $$E(u)\;:\;=\frac{1}{2}\sum_{i}u_{i}^{2}-\frac{1}{2}\sum_{i\;\neq \;j}W_{ij}\rho (u_{i})\rho (u_{j})-\sum_{i}b_{i}\rho (u_{i}).$$

**Figure 1:**
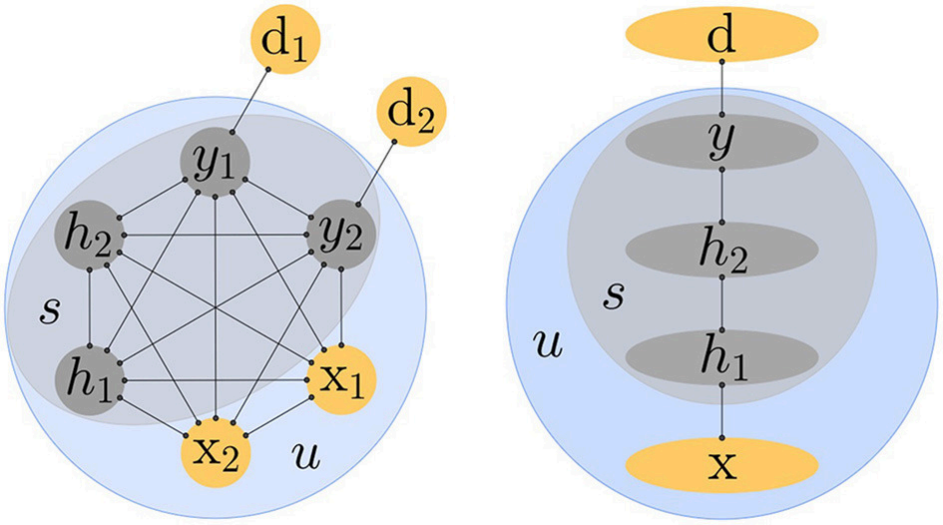
**The input units *x* are always clamped**. The state variable *s* includes the hidden units *h* and output units *y*. The targets are denoted by *d*. The network is recurrently connected with symmetric connections. **Left**. Equilibrium Propagation applies to any architecture, even a fully connected network. **Right**. The connection with Backpropagation is more obvious when the network has a layered architecture.

Note that the network is recurrently connected with symmetric connections, that is *W*_*ij*_ = *W*_*ji*_. The algorithm presented here is applicable to any architecture (so long as connections are symmetric), even a fully connected network. However, to make the connection to backpropagation more obvious, we will consider more specifically a layered architecture with no skip-layer connections and no lateral connections within a layer (Figure 1).

In the supervised setting studied here, the units of the network are split in three sets: the inputs *x* which are always clamped (just like in other models such as the conditional Boltzmann machine), the hidden units *h* (which may themselves be split in several layers) and the output units *y*. We use the notation *d* for the targets, which should not be confused with the outputs *y*. The set of all units in the network is *u* = {*x, h, y*}. As usual in the supervised learning scenario, the output units *y* aim to replicate their targets *d*. The discrepancy between the output units *y* and the targets *d* is measured by the quadratic cost function:

(2) $$C\;:\;=\frac{1}{2}\|y-d{\|}^{2}.$$

The cost function *C* also acts as an external potential energy for the output units, which can drive them toward their target. A novelty in our work, with respect to previously studied energy-based models, is that we introduce the “total energy function” *F*, which takes the form:

(3) F : =E+βC,

where β ≥ 0 is a real-valued scalar that controls whether the output *y* is pushed toward the target *d* or not, and by how much. We call β the “influence parameter” or “clamping factor.” The total energy *F* is the sum of two potential energies: the internal potential *E* that models the interactions within the network, and the external potential β*C* that models how the targets influence the output units. Contrary to Boltzmann Machines where the visible units are either free or (fully) clamped, here the real-valued parameter β allows the output units to be *weakly clamped*.

### 2.2. The neuronal dynamics

We denote the state variable of the network by *s* = {*h, y*} which does not include the input units *x* since they are always clamped. We assume that the time evolution of the state variable *s* is governed by the gradient dynamics:

(4) $$\frac{ds}{dt}=-\frac{\partial F}{\partial s}.$$

Unlike more conventional artificial neural networks, the model studied here is a continuous-time dynamical system described by the differential equation of motion (Equation 4). The total energy of the system decreases as time progresses (θ, β, *x*, and *d* being fixed) since:

(5) $$\frac{dF}{dt}=\frac{\partial F}{\partial s}\cdot \frac{ds}{dt}=-{\left\|\frac{ds}{dt}\right\|}^{2}\leq 0.$$

The energy stops decreasing when the network has reached a fixed point:

(6) $$\frac{dF}{dt}=0\;\Leftrightarrow \;\frac{ds}{dt}=0\;\Leftrightarrow \;\frac{\partial F}{\partial s}=0.$$

The differential equation of motion (Equation 4) can be seen as a sum of two “forces” that act on the temporal derivative of *s*:

(7) $$\frac{ds}{dt}=-\frac{\partial E}{\partial s}-\beta \frac{\partial C}{\partial s}.$$

The “internal force” induced by the internal potential (the Hopfield energy, Equation 1) on the *i*-th unit is:

(8) $$-\frac{\partial E}{\partial s_{i}}=\rho \prime (s_{i})\left(\sum_{j\neq i}W_{ij}\rho (u_{j})+b_{i}\right)-s_{i},$$

while the “external force” induced by the external potential (Equation 2) on *h*_*i*_ and *y*_*i*_ is, respectively:

(9) $$-\beta \frac{\partial C}{\partial h_{i}}=0\text{ and }-\beta \frac{\partial C}{\partial y_{i}}=\beta (d_{i}-y_{i}).$$

The form of Equation (8) is reminiscent of a leaky integrator neuron model, in which neurons are seen as performing leaky temporal integration of their past inputs. Note that the hypothesis of symmetric connections (*W*_*ij*_ = *W*_*ji*_) was used to derive Equation (8). As discussed in Bengio and Fischer (2015), the factor $\rho \prime (s_{i})$ would suggest that when a neuron is saturated [firing at the maximal or minimal rate so that $\rho \prime (s_{i})\approx 0$], its state is not sensitive to external inputs, while the leaky term drives it out of the saturation regime, toward its resting value *s*_*i*_ = 0.

The form of Equation (9) suggests that when β = 0, the output units are not sensitive to the outside world *d*. In this case, we say that the network is in the *free phase* (or first phase). On the contrary, when β > 0, the “external force” drives the output unit *y*_*i*_ toward the target *d*_*i*_. In this case, we say that the network is in the *weakly clamped phase* (or second phase).

Finally, a more likely dynamics would include some form of noise. The notion of fixed point is then replaced by that of stationary distribution. In Appendix C, we present a stochastic framework that naturally extends the analysis presented here.

### 2.3. Free phase, weakly clamped phase, and backpropagation of errors

In the first phase of training, the inputs are clamped and β = 0 (the output units are free). We call this phase the *free phase* and the state toward which the network converges is the *free fixed point* *u*^0^. The prediction is read out on the output units *y* at the fixed point.

In the second phase (which we call *weakly clamped phase*), the influence parameter β is changed to a small positive value β > 0 and the novel term β*C* added to the energy function (Equation 3) induces a new “external force” that acts on the output units (Equation 9). This force models the observation of *d*: it nudges the output units from their free fixed point value in the direction of their target. Since this force only acts on the output units, the hidden units are initially at equilibrium at the beginning of the weakly clamped phase, but the perturbation caused at the output units will propagate in the hidden units as time progresses. When the architecture is a multi-layered net (Figure 1, Right), the perturbation at the output layer propagates backwards across the hidden layers of the network. This propagation is thus better thought of as a “back-propagation.” The net eventually settles to a new fixed point (corresponding to the new positive value of β) which we call *weakly clamped fixed point* and denote by *u*^β^.

Remarkably, the perturbation that is (back-)propagated during the second phase corresponds to the propagation of error derivatives. It was first shown by Bengio and Fischer (2015) that, starting from the free fixed point, the early changes of neural activities during the weakly clamped phase (caused by the output units moving toward their target) approximate some kind of error derivatives with respect to the layers' activities. They considered a regular multi-layer neural network with no skip-layer connections and no lateral connections within a layer.

In this paper, we show that the weakly clamped phase also implements the (back)-propagation of error derivatives with respect to the synaptic weights. In the limit β → 0, the update rule:

(10) $$\Delta W_{ij}\propto \frac{1}{\beta }\left(\rho \left(u_{i}^{\beta }\right)\rho \left(u_{j}^{\beta }\right)-\rho \left(u_{i}^{0}\right)\rho \left(u_{j}^{0}\right)\right)$$

gives rise to stochastic gradient descent on $J\;:=\frac{1}{2}||y^{0}-d|{|}^{2}$, where *y*^0^ is the state of the output units at the free fixed point. We will state and prove this theorem in a more general setting in Section 3. In particular, this result holds for any architecture and not just a layered architecture (Figure 1) like the one considered by Bengio and Fischer (2015).

The learning rule (Equation 10) is a kind of contrastive Hebbian learning rule, somewhat similar to the one studied by Movellan (1990) and the Boltzmann machine learning rule. The differences with these algorithms will be discussed in Section 4.

We call our learning algorithm Equilibrium Propagation. In this algorithm, leaky integrator neural computation (as described in Section 2.2), performs both *inference* (in the free phase), and *error back-propagation* (in the weakly clamped phase).

### 2.4. Connection to spike-timing dependent plasticity

Spike-Timing Dependent Plasticity (STDP) is believed to be a prominent form of synaptic change in neurons (Markram and Sakmann, 1995; Gerstner et al., 1996), and see Markram et al. (2012) for a review.

The STDP observations relate the expected change in synaptic weights to the timing difference between post-synaptic and pre-synaptic spikes. This is the result of experimental observations in biological neurons, but its role as part of a learning algorithm remains a topic where more exploration is needed. Here, is an attempt in this direction.

Experimental results by Bengio et al. (2015b) show that if the temporal derivative of the synaptic weight *W*_*ij*_ satisfies:

(11) $$\frac{dW_{ij}}{dt}\propto \rho (u_{i})\frac{du_{j}}{dt},$$

then one recovers the experimental observations by Bi and Poo (2001) in biological neurons. Xie and Seung (2000) have studied the learning rule:

(12) $$\frac{dW_{ij}}{dt}\propto \rho (u_{i})\frac{d\rho (u_{j})}{dt}.$$

Note, that the two rules (Equations 11, 12) are the same up to a factor $\rho \prime (u_{j})$. An advantage of Equation (12) is that it leads to a more natural view of the update rule in the case of tied weights studied here (*W*_*ij*_ = *W*_*ji*_). Indeed, the update should take into account the pressures from both the *i* to *j* and *j* to *i* synapses, so that the total update under constraint is:

(13) $$\frac{dW_{ij}}{dt}\propto \rho (u_{i})\frac{d\rho (u_{j})}{dt}+\rho (u_{j})\frac{d\rho (u_{i})}{dt}=\frac{d}{dt}\rho (u_{i})\rho (u_{j}).$$

By integrating Equation (13) on the path from the free fixed point *u*^0^ to the weakly clamped fixed point *u*^β^ during the second phase, we get:

(14) $$\begin{array}{l} \Delta W_{ij}\;\propto \;\int \frac{dW_{ij}}{dt}dt=\int \frac{d}{dt}\rho (u_{i})\rho (u_{j})dt=\int d\left(\rho (u_{i})\rho (u_{j})\right)\\ =\rho \left(u_{i}^{\beta }\right)\rho \left(u_{j}^{\beta }\right)-\rho \left(u_{i}^{0}\right)\rho \left(u_{j}^{0}\right), \end{array}$$

which is the same learning rule as Equation (10) up to a factor 1/β. Therefore, the update rule (Equation 10) can be interpreted as a continuous-time integration of Equation (12), in the case of symmetric weights, on the path from *u*^0^ to *u*^β^ during the second phase.

We propose two possible interpretations for the synaptic plasticity in our model.

**First view**. In the first phase, a anti-Hebbian update occurs at the free fixed point $\Delta W_{ij}\propto -\;\rho (u_{i}^{0})\rho (u_{j}^{0})$. In the second phase, a Hebbian update occurs at the weakly-clamped fixed point $\Delta W_{ij}\propto +\;\rho (u_{i}^{\beta })\rho (u_{j}^{\beta })$.

**Second view**. In the first phase, no synaptic update occurs: Δ*W*_*ij*_ = 0. In the second phase, when the neurons' state move from the free fixed point *u*^0^ to the weakly-clamped fixed point *u*^β^, the synaptic weights follow the “tied version” of the continuous-time update rule $\frac{dW_{ij}}{dt}\propto \rho \left(u_{i}\right)\frac{d\rho \left(u_{j}\right)}{dt}+\rho \left(u_{j}\right)\frac{d\rho \left(u_{i}\right)}{dt}$.

## 3. A machine learning framework for energy based models

In this section we generalize the setting presented in Section 2. We lay down the basis for a new machine learning framework for energy-based models, in which Equilibrium Propagation plays a role analog to Backpropagation in computational graphs to compute the gradient of an objective function. Just like the Multi Layer Perceptron is the prototype of computational graphs in which Backpropagation is applicable, the continuous Hopfield model presented in Section 2 appears to be the prototype of models which can be trained with Equilibrium Propagation.

In our new machine learning framework, the central object is the total energy function *F*: all quantities of interest (fixed points, cost function, objective function, gradient formula) can be defined or formulated directly in terms of *F*.

Besides, in our framework, the “prediction” (or fixed point) is defined *implicitly* in terms of the data point and the parameters of the model, rather than *explicitly* (like in a computational graph). This implicit definition makes applications on digital hardware (such as GPUs) less practical as it needs long inference phases involving numerical optimization of the energy function. But we expect that this framework could be very efficient if implemented by analog circuits, as suggested by Hertz et al. (1997).

The framework presented in this section is deterministic, but a natural extension to the stochastic case is presented in Appendix C.

### 3.1. Training objective

In this section, we present the general framework while making sure to be consistent with the notations and terminology introduced in Section 2. We denote by *s* the state variable of the network, v the state of the external world (i.e., the data point being processed), and θ the set of free parameters to be learned. The variables *s*, v, and θ are real-valued vectors. The state variable *s* spontaneously moves toward low-energy configurations of an energy function *E*(θ, v, *s*). Besides that, a cost function *C*(θ, v, *s*) measures how “good” is a state is. The goal is to make low-energy configurations have low cost value.

For fixed θ and v, we denote by $s_{\theta ,\text{v}}^{0}$ a local minimum of *E*, also called fixed point, which corresponds to the “prediction” from the model:

(15) $$s_{\theta ,v}^{0}\in \underset{s}{\text{arg min}}\;E(\theta ,\text{v},s).$$

Here, we use the notation argmin to refer to the set of local minima. The objective function that we want to optimize is:

(16) $$J(\theta ,\text{v})\;:\;=C\left(\theta ,\text{v},s_{\theta ,v}^{0}\right).$$

Note the distinction between the cost function *C* and the objective function *J*: the cost function is defined for any state *s*, whereas the objective function is the cost associated to the fixed point $s_{\theta ,\text{v}}^{0}$.

Now that the objective function has been introduced, we define the training objective (for a single data point v) as:

(17) $$\text{find }\underset{\theta }{\text{arg min}}\;J(\theta ,\text{v}).$$

A formula to compute the gradient of *J* will be given in Section 3.3 (Theorem 1). Equivalently, the training objective can be reformulated as the following constrained optimization problem:

(18) $$\begin{array}{rc} \text{find} & \;\underset{\theta ,s}{\text{arg min}}\;C\left(\theta ,v,s\right) \end{array}$$

(19) $$\begin{array}{rc} \text{subject to} & \;\frac{\partial E}{\partial s}\left(\theta ,v,s\right)=0, \end{array}$$

where the constraint $\frac{\partial E}{\partial s}\left(\theta ,v,s\right)=0$ is the fixed point condition. For completeness, a solution to this constrained optimization problem is given in Appendix B as well. Of course, both formulations of the training objective lead to the same gradient update on θ.

Note that, since the cost *C*(θ, v, *s*) may depend on θ, it can include a regularization term of the form λΩ(θ), where Ω(θ) is a *L*_1_ or *L*_2_ norm penalty for example.

In Section 2 we had *s* = {*h, y*} for the state variable, v = {*x, d*} for the state of the outside world, θ = (*W, b*) for the set of learned parameters, and the energy function *E* and cost function *C* were of the form *E*(θ, v, *s*) = *E*(θ, *x, h, y*) and *C*(θ, v, *s*) = *C*(*y, d*).

### 3.2. Total energy function

Following Section 2, we introduce the total energy function:

(20) F(θ,v,β,s) : = E(θ,v,s)+β C(θ,v,s),

where β is a real-valued scalar called “influence parameter.” Then we extend the notion of fixed point for any value of β. The fixed point (or energy minimum), denoted by $s_{\theta ,\text{v}}^{\beta }$, is characterized by:

(21) $$\frac{\partial F}{\partial s}\left(\theta ,\text{v},\beta ,s_{\theta ,\text{v}}^{\beta }\right)=0$$

and $\frac{\partial^{2}F}{\partial s^{2}}(\theta ,\text{v},\beta ,s_{\theta ,v}^{\beta })$ is a symmetric positive definite matrix. Under mild regularity conditions on *F*, the implicit function theorem ensures that, for fixed v, the funtion $\left(\theta ,\beta \right)\mapsto s_{\theta ,\text{v}}^{\beta }$ is differentiable.

### 3.3. The learning algorithm: equilibrium propagation

**Theorem 1** (Deterministic version). *The gradient of the objective function with respect to θ is given by the formula*:

(22) $$\frac{\partial J}{\partial \theta }(\theta ,\text{v})=\underset{\beta \to 0}{\lim}\frac{1}{\beta }\left(\frac{\partial F}{\partial \theta }\left(\theta ,\text{v},\beta ,s_{\theta ,\text{v}}^{\beta }\right)-\frac{\partial F}{\partial \theta }\left(\theta ,\text{v},0,s_{\theta ,\text{v}}^{0}\right)\right),$$

*or equivalently*

(23) $$\frac{\partial J}{\partial \theta }(\theta ,\text{v})=\frac{\partial C}{\partial \theta }\left(\theta ,\text{v},s_{\theta ,\text{v}}^{0}\right)+\underset{\beta \to 0}{\lim}\frac{1}{\beta }(\frac{\partial E}{\partial \theta }\left(\theta ,\text{v},s_{\theta ,\text{v}}^{\beta }\right)-\frac{\partial E}{\partial \theta }\left(\theta ,\text{v},s_{\theta ,\text{v}}^{0}\right)).$$

Theorem 1 will be proved in Appendix A. Note that the parameter β in Theorem 1 need not be positive (We only need β → 0). Using the terminology introduced in Section 2, we call $s_{\theta ,\text{v}}^{0}$ the free fixed point, and $s_{\theta ,\text{v}}^{\beta }$ the nudged fixed point (or weakly-clamped fixed point in the case β > 0). Moreover, we call a free phase (resp. nudged phase or weakly-clamped phase) a procedure that yields a free fixed point (resp. nudged fixed point or weakly-clamped fixed point) by minimizing the energy function *F* with respect to *s*, for β = 0 (resp. β ≠ 0). Theorem 1 suggests the following two-phase training procedure. Given a data point v:

1. Run a free phase until the system settles to a free fixed point $s_{\theta ,\text{v}}^{0}$ and collect $\frac{\partial F}{\partial \theta }\left(\theta ,v,0,s_{\theta ,\text{v}}^{0}\right)$.
2. Run a nudged phase for some β ≠ 0 such that |β| is “small,” until the system settles to a nudged fixed point $s_{\theta ,\text{v}}^{\beta }$ and collect $\frac{\partial F}{\partial \theta }\left(\theta ,v,\beta ,s_{\theta ,\text{v}}^{\beta }\right)$.
3. Update the parameter θ according to
  (24) $$\Delta \theta \propto -\frac{1}{\beta }\left(\frac{\partial F}{\partial \theta }\left(\theta ,\text{v},\beta ,s_{\theta ,\text{v}}^{\beta }\right)-\frac{\partial F}{\partial \theta }\left(\theta ,\text{v},0,s_{\theta ,\text{v}}^{0}\right)\right).$$

Consider the case β > 0. Starting from the free fixed point $s_{\theta ,\text{v}}^{0}$ (which corresponds to the “prediction”), a small change of the parameter β (from the value β = 0 to a value β > 0) causes slight modifications in the interactions in the network. This small perturbation makes the network settle to a nearby weakly-clamped fixed point $s_{\theta ,\text{v}}^{\beta }$. Simultaneously, a kind of contrastive update rule for θ is happening, in which the energy of the free fixed point is increased and the energy of the weakly-clamped fixed point is decreased. We call this learning algorithm Equilibrium Propagation.

Note that in the setting introduced in Section 2.1 the total energy function (Equation 3) is such that $\frac{\partial F}{\partial W_{ij}}=-\rho \left(u_{i}\right)\rho \left(u_{j}\right)$, in agreement with Equation (10). In the weakly clamped phase, the novel term $\frac{1}{2}\beta ||y-d|{|}^{2}$ added to the energy *E* (with β > 0) slightly attracts the output state *y* to the target *d*. Clearly, the weakly clamped fixed point is better than the free fixed point in terms of prediction error. The following proposition generalizes this property to the general setting.

**Proposition 2** (Deterministic version). *The derivative of the function*

(25) $$\beta \mapsto C\left(\theta ,\text{v},s_{\theta ,\text{v}}^{\beta }\right)$$

*at β = 0 is non-positive*.

Proposition 2 will also be proved in Appendix A. This proposition shows that, unless the free fixed point $s_{\theta ,\text{v}}^{0}$ is already optimal in terms of cost value, for β > 0 small enough, the weakly-clamped fixed point $s_{\theta ,\text{v}}^{\beta }$ achieves lower cost value than the free fixed point. Thus, a small perturbation due to a small increment of β would nudge the system toward a state that reduces the cost value. This property sheds light on the update rule (Theorem 1), which can be seen as a kind of contrastive learning rule (somehow similar to the Boltzmann machine learning rule) where we *learn* (make more probable) the slightly better state $s_{\theta ,\text{v}}^{\beta }$ by reducing its energy and *unlearn* (make less probable) the slightly worse state $s_{\theta ,\text{v}}^{0}$ by increasing its energy.

However, our learning rule is different from the Boltzmann machine learning rule and the contrastive Hebbian learning rule. The differences between these algorithms will be discussed in section 4.

### 3.4. Another view of the framework

In Sections 3.1 and 3.2 (as well as in Section 2) we first defined the energy function *E* and the cost function *C*, and then we introduced the total energy *F* : = *E* + β*C*. Here, we propose an alternative view of the framework, where we reverse the order in which things are defined.

Given a total energy function *F* (which models all interactions within the network as well as the action of the external world on the network), we can define all quantities of interest in terms of *F*. Indeed, we can define the energy function *E* and the cost function *C* as:

(26) $$E(\theta ,\text{v},s)\;:\;=F\left(\theta ,\text{v},0,s\right)\text{ and }C(\theta ,\text{v},s)\;:\;=\frac{\partial F}{\partial \beta }\left(\theta ,\text{v},0,s\right),$$

where *F* and $\frac{\partial F}{\partial \beta }$ are evaluated with the argument β set to 0. Obviously the fixed points $s_{\theta ,\text{v}}^{0}$ and $s_{\theta ,\text{v}}^{\beta }$ are directly defined in terms of *F*, and so is the objective function $J(\theta ,\text{v})\;:=C(\theta ,\text{v},s_{\theta ,\text{v}}^{0})$. The learning algorithm (Theorem 1) is also formulated in terms of *F*^3^. From this perspective, *F* contains all the information about the model and can be seen as the central object of the framework. For instance, the cost *C* represents the marginal variation of the total energy *F* due to a change of β.

As a comparison, in the traditional framework for Deep Learning, a model is represented by a (differentiable) computational graph in which each node is defined as a function of its parents. The set of functions that define the nodes fully specifies the model. The last node of the computational graph represents the cost to be optimized, while the other nodes represent the state of the layers of the network, as well as other intermediate computations.

In the framework for machine learning proposed here (the framework suited for Equilibrium Propagation), the analog of the set of functions that define the nodes in the computational graph is the total energy function *F*.

### 3.5. Backpropagation vs. equilibrium propagation

**Figure 2:**
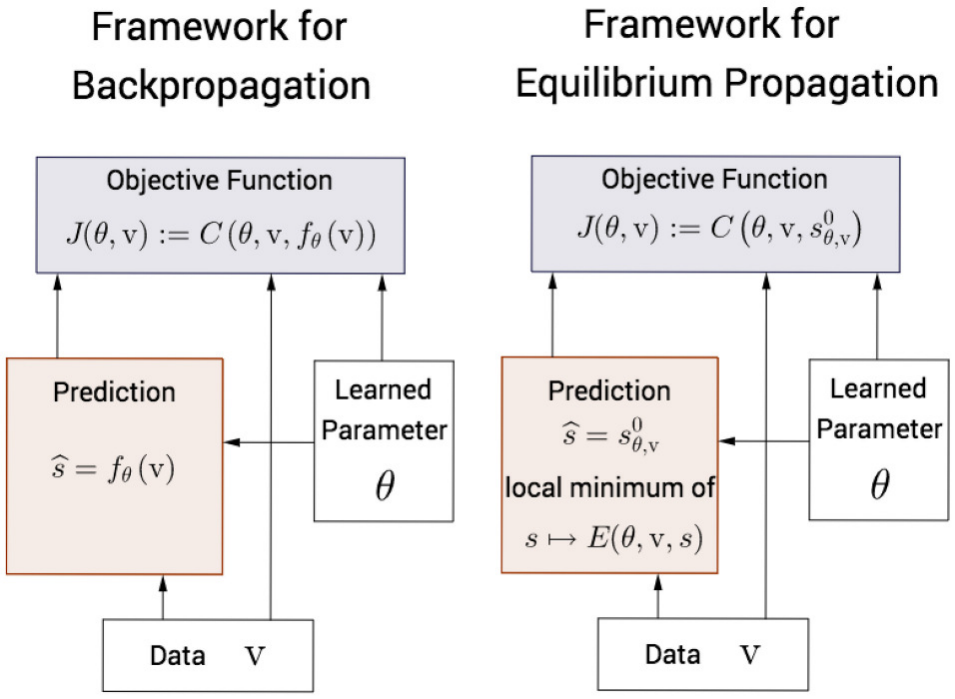
**Comparison between the traditional framework for Deep Learning and our framework**. **Left**. In the traditional framework, the state of the network *f*_θ_(v) and the objective function *J*(θ, v) are *explicit* functions of θ and v and are computed *analytically*. The gradient of the objective function is also computed analytically thanks to the Backpropagation algorithm (a.k.a automatic differentiation). **Right**. In our framework, the free fixed point $s_{\theta ,\text{v}}^{0}$ is an *implicit* function of θ and v and is computed *numerically*. The nudged fixed point $s_{\theta ,\text{v}}^{\beta }$ and the gradient of the objective function are also computed numerically, following our learning algorithm: Equilibrium Propagation.

In the traditional framework for Deep Learning (Figure 2, left), each node in the computational graph is an *explicit* differentiable function of its parents. The state of the network $\hat{s}=f_{\theta }\left(v\right)$ and the objective function *J*(θ, v) = *C*(θ, v, *f*_θ_(v)) are computed *analytically*, as functions of θ and v, in the forward pass. The Backpropagation algorithm (a.k.a automatic differentiation) enables to compute the error derivatives analytically too, in the backward pass. Therefore, the state of the network $\hat{s}=f_{\theta }\left(v\right)$ (forward pass) and the gradient of the objective function $\frac{\partial J}{\partial \theta }\left(\theta ,v\right)$ (backward pass) can be computed *efficiently* and *exactly*^4^.

In the framework for machine learning that we propose here (Figure 2, right), the free fixed point $\hat{s}=s_{\theta ,\text{v}}^{0}$ is an *implicit* function of θ and v, characterized by $\frac{\partial E}{\partial s}(\theta ,\text{v},s_{\theta ,\text{v}}^{0})=0$. The free fixed point is computed *numerically*, in the free phase (first phase). Similarly the nudged fixed point $s_{\theta ,\text{v}}^{\beta }$ is an implicit function of θ, v, and β, and is computed numerically in the nudged phase (second phase). Equilibrium Propagation *estimates* (for the particular value of β chosen in the second phase) the gradient of the objective function $\frac{\partial J}{\partial \theta }\left(\theta ,v\right)$ based on these two fixed points. The requirement for numerical optimization in the first and second phases make computations *inefficient* and *approximate*. The experiments in Section 5 will show that the free phase is fairly long when performed with a discrete-time computer simulation. However, we expect that the full potential of the proposed framework could be exploited on analog hardware (instead of digital hardware), as suggested by Hertz et al. (1997).

## 4. Related work

In Section 2.3, we have discussed the relationship between Equilibrium Propagation and Backpropagation. In the weakly clamped phase, the change of the influence parameter β creates a perturbation at the output layer which propagates backwards in the hidden layers. The error derivatives and the gradient of the objective function are encoded by this perturbation.

In this section, we discuss the connection between our work and other algorihms, starting with Contrastive Hebbian Learning. Equilibrium Propagation offers a new perspective on the relationship between Backpropagation in feedforward nets and Contrastive Hebbian Learning in Hopfield nets and Boltzmann machines (Table 1).

**Table 1:** **Correspondence of the phases for different learning algorithms: Back-propagation, Equilibrium Propagation (our algorithm), Contrastive Hebbian Learning (and Boltzmann Machine Learning) and Almeida-Pineida's Recurrent Back-Propagation**.

	**Backprop**	**Equilibrium Prop**	**Contrastive Hebbian Learning**	**Almeida-Pineida**
First Phase	Forward Pass	Free Phase	Free Phase (or Negative Phase)	Free Phase
Second Phase	Backward Pass	Weakly Clamped Phase	Clamped Phase (or Positive Phase)	Recurrent Backprop

### 4.1. Link to contrastive hebbian learning

Despite the similarity between our learning rule and the Contrastive Hebbian Learning rule (CHL) for the continuous Hopfield model, there are important differences.

First, recall that our learning rule is:

(27) $$\Delta W_{ij}\propto \underset{\beta \to 0}{\lim}\frac{1}{\beta }\left(\rho \left(u_{i}^{\beta }\right)\rho \left(u_{j}^{\beta }\right)-\rho \left(u_{i}^{0}\right)\rho \left(u_{j}^{0}\right)\right),$$

where *u*^0^ is the free fixed point and *u*^β^ is the *weakly* clamped fixed point. The Contrastive Hebbian Learning rule is:

(28) $$\Delta W_{ij}\propto \rho \left(u_{i}^{\infty }\right)\rho \left(u_{j}^{\infty }\right)-\rho \left(u_{i}^{0}\right)\rho \left(u_{j}^{0}\right),$$

where *u*^∞^ is the *fully* clamped fixed point (i.e., fixed point with fully clamped outputs). We choose the notation *u*^∞^ for the fully clamped fixed point because it corresponds to β → +∞ with the notations of our model. Indeed Equation (9) shows that in the limit β → +∞, the output unit *y*_*i*_ moves infinitely fast toward *y*_*i*_, so *y*_*i*_ is immediately clamped to *y*_*i*_ and is no longer sensitive to the “internal force” (Equation 8). Another way to see it is by considering Equation (3): as β → +∞, the only value of *y* that gives finite energy is *d*.

The objective functions that these two algorithms optimize also differ. Recalling the form of the Hopfield energy (Equation 1) and the cost function (Equation 2), Equilibrium Propagation computes the gradient of:

(29) $$J=\frac{1}{2}\left\|y^{0}-d^{2}\right\|,$$

where *y*^0^ is the output state at the free phase fixed point *u*^0^, while CHL computes the gradient of:

(30) $$J_{\text{CHL}}=E\left(u^{\infty }\right)-E\left(u^{0}\right).$$

The objective function for CHL has theoretical problems: it may take negative values if the clamped phase and free phase stabilize in different modes of the energy function, in which case the weight update is inconsistent and learning usually deteriorates, as pointed out by Movellan (1990). Our objective function does not suffer from this problem, because it is defined in terms of local perturbations, and the implicit function theorem guarantees that the weakly clamped fixed point will be close to the free fixed point (thus in the same mode of the energy function).

We can also reformulate the learning rules and objective functions of these algorithms using the notations of the general setting (Section 3). For Equilibrium Propagation we have:

$$\Delta \theta \propto -\underset{\beta \to 0}{\lim}\frac{1}{\beta }\left(\frac{\partial F}{\partial \theta }\left(\theta ,\text{v},\beta ,s_{\theta ,\text{v}}^{\beta }\right)-\frac{\partial F}{\partial \theta }\left(\theta ,\text{v},0,s_{\theta ,\text{v}}^{0}\right)\right)$$

and

(31) $$J(\theta ,\text{v})=\frac{\partial F}{\partial \beta }\left(\theta ,\text{v},0,s_{\theta ,\text{v}}^{0}\right).$$

As for Contrastive Hebbian Learning, one has

$$\Delta \theta \propto -\left(\frac{\partial F}{\partial \theta }\left(\theta ,\text{v},\infty ,s_{\theta ,\text{v}}^{\infty }\right)-\frac{\partial F}{\partial \theta }\left(\theta ,\text{v},0,s_{\theta ,\text{v}}^{0}\right)\right)$$

and

(32) $$J_{\text{CHL}}(\theta ,\text{v})=F(\theta ,\text{v},\infty ,s_{\theta ,\text{v}}^{\infty })-F(\theta ,\text{v},0,s_{\theta ,\text{v}}^{0}),$$

where β = 0 and β = ∞ are the values of β corresponding to free and (fully) clamped outputs, respectively.

Our learning algorithm is also more flexible because we are free to choose the cost function *C* (as well as the energy funtion *E*), whereas the contrastive function that CHL optimizes is fully determined by the energy function *E*.

### 4.2. Link to boltzmann machine learning

Again, the log-likelihood that the Boltzmann machine optimizes is determined by the Hopfield energy *E*, whereas we have the freedom to choose the cost function in the framework for Equilibrium Propagation.

As discussed in Section 2.3, the second phase of Equilibrium Propagation (going from the free fixed point to the weakly clamped fixed point) can be seen as a brief “backpropagation phase” with weakly clamped target outputs. By contrast, in the positive phase of the Boltzmann machine, the target is fully clamped, so the (correct version of the) Boltzmann machine learning rule requires two separate and independent phases (Markov chains), making an analogy with backprop less obvious.

Our algorithm is also similar in spirit to the CD algorithm (Contrastive Divergence) for Boltzmann machines. In our model, we start from a free fixed point (which requires a long relaxation in the free phase) and then we run a short weakly clamped phase. In the CD algorithm, one starts from a positive equilibrium sample with the visible units clamped (which requires a long positive phase Markov chain in the case of a general Boltzmann machine) and then one runs a short negative phase. But there is an important difference: our algorithm computes the *correct* gradient of our objective function (in the limit β → 0), whereas the CD algorithm computes a *biased estimator* of the gradient of the log-likelihood. The CD_1_ update rule is provably not the gradient of any objective function and may cycle indefinitely in some pathological cases (Sutskever and Tieleman, 2010).

Finally, in the supervised setting presented in Section 2, a more subtle difference with the Boltzmann machine is that the “output” state *y* in our model is best thought of as being part of the latent state variable *s*. If we were to make an analogy with the Boltzmann machine, the visible units of the Boltzmann machine would be v = {*x, d*}, while the hidden units would be *s* = {*h, y*}. In the Boltzmann machine, the state of the external world is inferred directly on the visible units (because it is a probabilistic generative model that maximizes the log-likelyhood of the data), whereas in our model we make the choice to integrate in *s* special latent variables *y* that aim to match the target *d*.

### 4.3. Link to recurrent back-propagation

Directly connected to our model is the work by Pineda (1987) and Almeida (1987) on recurrent back-propagation. They consider the same objective function as ours, but formulate the problem as a constrained optimization problem. In Appendix B, we derive another proof for the learning rule (Theorem 1) with the Lagrangian formalism for constrained optimization problems. The beginning of this proof is in essence the same as the one proposed by Pineda (1987); Almeida (1987), but there is a major difference when it comes to solving Equation (75) for the costate variable λ^*^. The method proposed by Pineda (1987) and Almeida (1987) is to use Equation (75) to compute λ^*^ by a fixed point iteration in a linearized form of the recurrent network. The computation of λ^*^ corresponds to their second phase, which they call *recurrent back-propagation*. However, this second phase does not follow the same kind of dynamics as the first phase (the free phase) because it uses a linearization of the neural activation rather than the fully non-linear activation^5^. From a biological plausibility point of view, having to use a different kind of hardware and computation for the two phases is not satisfying.

By contrast, like the continuous Hopfield net and the Boltzmann machine, our model involves only one kind of neural computations for both phases.

### 4.4. The model by Xie and Seung

Previous work on the back-propagation interpretation of contrastive Hebbian learning was done by Xie and Seung (2003).

The model by Xie and Seung (2003) is a modified version of the Hopfield model. They consider the case of a layered MLP-like network, but their model can be extended to a more general connectivity, as shown here. In essence, using the notations of our model (Section 2), the energy function that they consider is:

(33) $$E_{X\&S}(u)\;:\;=\frac{1}{2}\sum_{i}\;\gamma^{i}u_{i}^{2}-\sum_{i<j}\;\gamma^{j}W_{ij}\rho (u_{i})\rho (u_{j})-\sum_{i}\;\gamma^{i}b_{i}\rho (u_{i}).$$

The difference with Equation (1) is that they introduce a parameter γ, assumed to be small, that scales the strength of the connections. Their update rule is the contrastive Hebbian learning rule which, for this particular energy function, takes the form:

(34) $$\begin{array}{l} \Delta W_{ij}\propto -\left(\frac{\partial E_{X\&S}}{\partial W_{ij}}\left(u^{\infty }\right)-\frac{\partial E_{X\&S}}{\partial W_{ij}}\left(u^{0}\right)\right)\\ \;=\gamma^{j}\left(\rho \left(u_{i}^{\infty }\right)\rho \left(u_{j}^{\infty }\right)-\rho \left(u_{i}^{0}\right)\rho \left(u_{j}^{0}\right)\right) \end{array}$$

for every pair of indices (*i, j*) such that *i* < *j*. Here, *u*^∞^ and *u*^0^ are the (fully) clamped fixed point and free fixed point, respectively. Xie and Seung (2003) show that in the regime γ → 0 this contrastive Hebbian learning rule is equivalent to back-propagation. At the free fixed point *u*^0^, one has $\frac{\partial E_{X\&S}}{\partial s_{i}}(u^{0})=0$ for every unit *s*_*i*_^6^, which yields, after dividing by γ*i* and rearranging the terms:

(35) $$s_{i}^{0}=\rho \prime \left(s_{i}^{0}\right)\left(\sum_{j\;<\;i}W_{ij}\rho \left(u_{j}^{0}\right)+\sum_{j>i}\gamma^{j\;-\;i}W_{ij}\rho \left(u_{j}^{0}\right)+b_{i}\right).$$

In the limit γ → 0, one gets $s_{i}^{0}\approx \rho \prime (s_{i}^{0})\left(\sum_{j<i}W_{ij}\rho (u_{j}^{0})+b_{i}\right)$, so that the network almost behaves like a feedforward net in this regime.

As a comparison, recall that in our model (Section 2) the energy function is:

(36) $$E(u)\;:\;=\frac{1}{2}\sum_{i}u_{i}^{2}-\sum_{i\;<\;j}W_{ij}\rho (u_{i})\rho (u_{j})-\sum_{i}b_{i}\rho (u_{i}),$$

the learning rule is:

(37) $$\begin{array}{l} \Delta W_{ij}\propto -\underset{\beta \to 0}{\lim}\frac{1}{\beta }\left(\frac{\partial E}{\partial W_{ij}}\left(u^{\beta }\right)-\frac{\partial E}{\partial W_{ij}}\left(u^{0}\right)\right)\\ \;=\underset{\beta \to 0}{\lim}\frac{1}{\beta }\left(\rho \left(u_{i}^{\beta }\right)\rho \left(u_{j}^{\beta }\right)-\rho \left(u_{i}^{0}\right)\rho \left(u_{j}^{0}\right)\right), \end{array}$$

and at the free fixed point, we have $\frac{\partial E}{\partial s_{i}}(u^{0})=0$ for every unit *s*_*i*_, which gives:

(38) $$s_{i}^{0}=\rho \prime \left(s_{i}^{0}\right)\left(\sum_{j\;\neq \;i}W_{ij}\rho \left(u_{j}^{0}\right)+b_{i}\right).$$

Here, are the main differences between our model and theirs. In our model, the feedforward and feedback connections are both strong. In their model, the feedback weights are tiny compared to the feedforward weights, which makes the (recurrent) computations look almost feedforward. In our second phase, the outputs are weakly clamped. In their second phase, they are fully clamped. The theory of our model requires a unique learning rate for the weights, while in their model the update rule for *W*_*ij*_ (with *i* < *j*) is scaled by a factor γ*j* (see Equation 34). Since γ is small, the learning rates for the weights vary on many orders of magnitude in their model. Intuitively, these multiple learning rates are required to compensate for the small feedback weights.

## 5. Implementation of the model and experimental results

In this section, we provide experimental evidence that our model described in Section 2 is trainable, by testing it on the classification task of MNIST digits (LeCun and Cortes, 1998). The MNIST dataset of handwritten digits consists of 60,000 training examples and 10,000 test examples. Each example *x* in the dataset is a gray-scale image of 28 by 28 pixels and comes with a label *d* ∈ {0, 1, …, 9}. We use the same notation *y* for the one-hot encoding of the target, which is a 10-dimensional vector.

**Figure 3:**
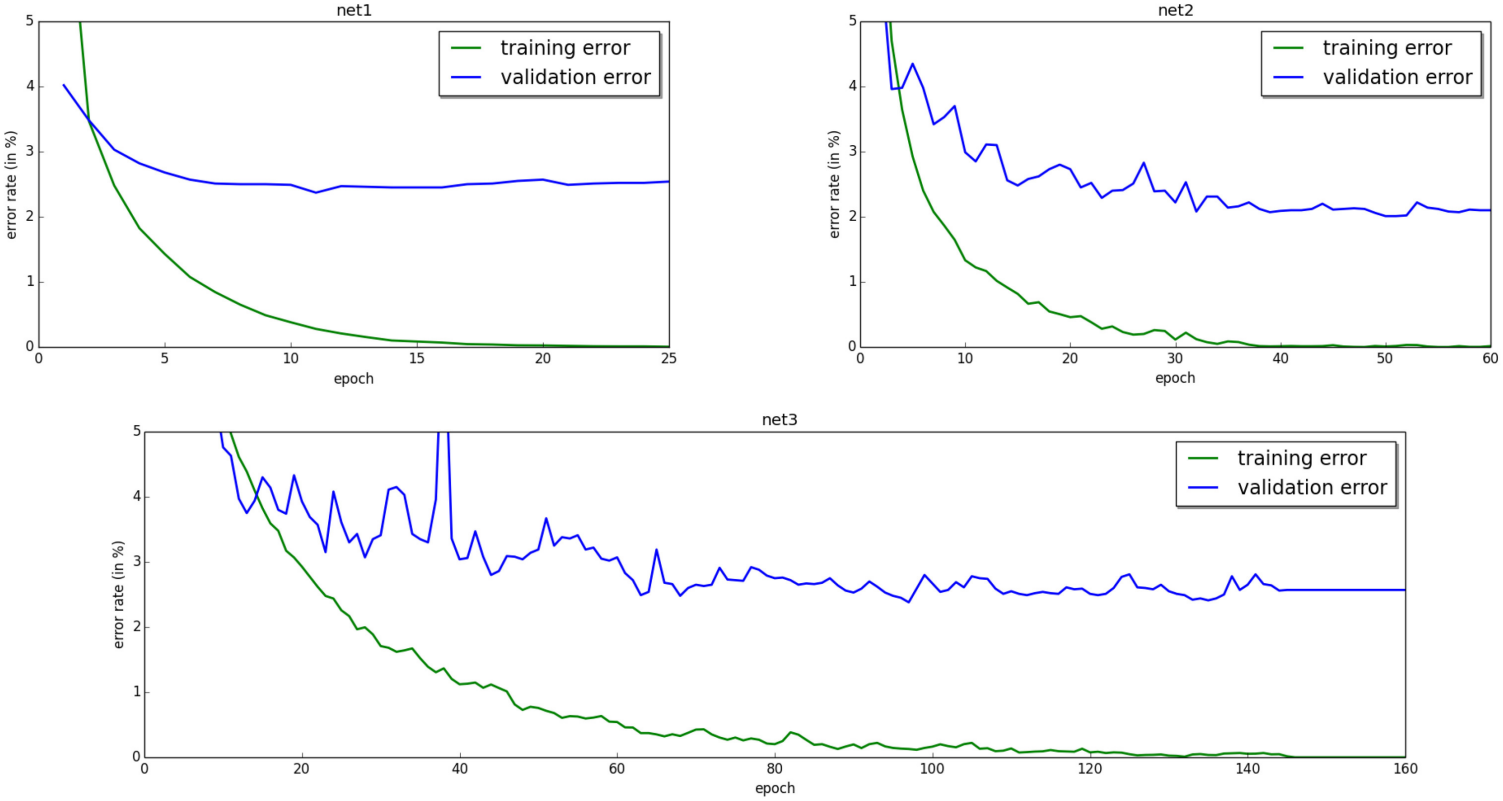
**Training and validation error for neural networks with one hidden layer of 500 units (top left)**, two hidden layers of 500 units **(top right)**, and three hidden layers of 500 units **(bottom)**. The training error eventually decreases to 0.00% in all three cases.

Recall that our model is a recurrently connected neural network with symmetric connections. Here, we train multi-layered networks with 1, 2, and 3 hidden layers, with no skip-layer connections and no lateral connections within layers. Although we believe that analog hardware would be more suited for our model, here we propose an implementation on digital hardware (a GPU). We achieve 0.00% training error. The generalization error lies between 2 and 3% depending on the architecture (Figure 3).

For each training example (*x, d*) in the dataset, training proceeds as follows:

1. Clamp *x*.
2. Run the free phase until the hidden and output units settle to the free fixed point, and collect $\rho \left(u_{i}^{0}\right)\rho \left(u_{j}^{0}\right)$ for every pair of units *i, j*.
3. Run the weakly clamped phase with a “small” β > 0 until the hidden and output units settle to the weakly clamped fixed point, and collect $\rho \left(u_{i}^{\beta }\right)\rho \left(u_{j}^{\beta }\right)$.
4. Update each synapse *W*_*ij*_ according to
  (39) $$\Delta W_{ij}\propto \frac{1}{\beta }\left(\rho \left(u_{i}^{\beta }\right)\rho \left(u_{j}^{\beta }\right)-\rho \left(u_{i}^{0}\right)\rho \left(u_{j}^{0}\right)\right).$$

The prediction is made at the free fixed point *u*^0^ at the end of the first phase relaxation. The predicted value *y*_pred_ is the index of the output unit whose activation is maximal among the 10 output units:

(40) $$y_{\text{pred}}\;:\;=\underset{i}{\text{arg max}}\;y_{i}^{0}.$$

Note that no constraint is imposed on the activations of the units of the output layer in our model, unlike more traditional neural networks where a softmax output layer is used to constrain them to sum up to 1. Recall that the objective function that we minimize is the square of the difference between our prediction and the one-hot encoding of the target value:

(41) $$J=\frac{1}{2}{\left\|d-y^{0}\right\|}^{2}.$$

### 5.1. Finite differences

#### 5.1.1. Implementation of the differential equation of motion

First we clamp *x*. Then the obvious way to implement Equation (4) is to discretize time into short time lapses of duration ϵ and to update each hidden and output unit *s*_*i*_ according to

(42) $$s_{i}\leftarrow s_{i}-\epsilon \frac{\partial F}{\partial s_{i}}(\theta ,\text{v},\beta ,s).$$

This is simply one step of gradient descent on the total energy *F*, with step size ϵ.

For our experiments, we choose the hard sigmoid activation function ρ(*s*_*i*_) = 0 ∨ *s*_*i*_ ∧ 1, where ∨ denotes the max and ∧ the min. For this choice of ρ, since $\rho \prime (s_{i})=0$ for *s*_*i*_ < 0, it follows from Equations (8) and (9) that if *h*_*i*_ < 0 then $\frac{\partial F}{\partial h_{i}}\left(\theta ,v,\beta ,s\right)=-h_{i}>0$. This force prevents the hidden unit *h*_*i*_ from going in the range of negative values. The same is true for the output units. Similarly, *s*_*i*_ cannot reach values above 1. As a consequence *s*_*i*_ must remain in the domain 0 ≤ *s*_*i*_ ≤ 1. Therefore, rather than the standard gradient descent (Equation 42), we will use a slightly different update rule for the state variable *s*:

(43) $$s_{i}\leftarrow 0\vee \left(s_{i}-\epsilon \frac{\partial F}{\partial s_{i}}(\theta ,\text{v},\beta ,s)\right)\wedge 1.$$

This little implementation detail turns out to be very important: if the *i*-th hidden unit was in some state *h*_*i*_ < 0, then Equation (42) would give the update rule *h*_*i*_ ← (1 − ϵ)*h*_*i*_, which would imply again *h*_*i*_ < 0 at the next time step (assuming ϵ < 1). As a consequence *h*_*i*_ would remain in the negative range forever.

#### 5.1.2. Choice of the step size ϵ

We find experimentally that the choice of ϵ has little influence as long as 0 < ϵ < 1. What matters more is the *total duration of the relaxation* Δ*t* = *n*_iter_×ϵ (where *n*_iter_ is the number of iterations). In our experiments we choose ϵ = 0.5 to keep *n*_iter_ = Δ*t*/ϵ as small as possible so as to avoid extra unnecessary computations.

#### 5.1.3. Duration of the free phase relaxation

We find experimentally that the number of iterations required in the free phase to reach the free fixed point is large and grows fast as the number of layers increases (Table 2), which considerably slows down training. More experimental and theoretical investigation would be needed to analyze the number of iterations required, but we leave that for future work.

**Table 2:** **Hyperparameters**.

**Architecture**	**Iterations**	**Iterations**	**ϵ**	**β**	**α_1_**	**α_2_**	**α_3_**	**α_4_**
	**(first phase)**	**(second phase)**						
784-500-10	20	4	0.5	1.0	0.1	0.05		
784-500-500-10	100	6	0.5	1.0	0.4	0.1	0.01	
784-500-500-500-10	500	8	0.5	1.0	0.128	0.032	0.008	0.002

*The learning rate ϵ is used for iterative inference (Equation 43). β is the value of the clamping factor in the second phase. α_k_ is the learning rate for updating the parameters in layer k*.

#### 5.1.4. Duration of the weakly clamped phase

During the weakly clamped phase, we observe that the relaxation to the weakly clamped fixed point is not necessary. We only need to “initiate” the movement of the units, and for that we use the following heuristic. Notice that the time constant of the integration process in the leaky integrator equation (Equation 8) is τ = 1. This time constant represents the time needed for a signal to propagate from a layer to the next one with “significant amplitude.” So the time needed for the error signals to back-propagate in the network is *Nτ* = *N*, where *N* is the number of layers (hiddens and output) of the network. Thus, we choose to perform *N*/ϵ iterations with step size ϵ = 0.5.

### 5.2. Implementation details and experimental results

To tackle the problem of the long free phase relaxation and speed-up the simulations, we use “persistent particles” for the latent variables to re-use the previous fixed point configuration for a particular example as a starting point for the next free phase relaxation on that example. This means that for each training example in the dataset, we store the state of the hidden layers at the end of the free phase, and we use this to initialize the state of the network at the next epoch. This method is similar in spirit to the PCD algorithm (Persistent Contrastive Divergence) for sampling from other energy-based models like the Boltzmann machine (Tieleman, 2008).

We find that it helps regularize the network if we choose the sign of β at random in the second phase. Note that the weight updates remain consistent thanks to the factor 1/β in the update rule $\Delta W_{ij}\propto \frac{1}{\beta }\left(\rho \left(u_{i}^{\beta }\right)\rho \left(u_{j}^{\beta }\right)-\rho \left(u_{i}^{0}\right)\rho \left(u_{j}^{0}\right)\right)$. Indeed, the left-derivative and the right-derivative of the function $\beta \mapsto \rho \left(u_{i}^{\beta }\right)\rho \left(u_{j}^{\beta }\right)$ at the point β = 0 coincide.

Although the theory presented in this paper requires a unique learning rate for all synaptic weights, in our experiments we need to choose different learning rates for the weight matrices of different layers to make the algorithm work. We do not have a clear explanation for this fact yet, but we believe that this is due to the finite precision with which we approach the fixed points. Indeed, the theory requires to be exactly at the fixed points, but in practice we minimize the energy function by numerical optimization, using Equation (43). The precision with which we approach the fixed points depends on hyperparameters such as the step size ϵ and the number of iterations *n*_iter_.

Let us denote by *h*_0_, *h*_1_, ⋯ , *h*_*N*_ the layers of the network (where *h*_0_ = *x* and *h*_*N*_ = *y*) and by *W*_*k*_ the weight matrix between the layers *h*_*k*−1_ and *h*_*k*_. We choose the learning rate α_*k*_ for *W*_*k*_ so that the quantities $\frac{\Delta W_{k}}{W_{k}}$ for *k* = 1, ⋯ , *N* are approximately the same in average (over training examples), where ||Δ*W*_*k*_|| represents the weight change of *W*_*k*_ after seeing a minibatch.

The hyperparameters chosen for each model are shown in Table 2 and the results are shown in Figure 3. We initialize the weights according to the Glorot-Bengio initialization (Glorot and Bengio, 2010). For efficiency of the experiments, we use minibatches of 20 training examples.

## 6. Discussion, looking forward

From a biological perspective, a troubling issue in the Hopfield model is the requirement of symmetric weights between the units. Note that the units in our model need not correspond exactly to actual neurons in the brain (it could be groups of neurons in a cortical microcircuit, for example). It remains to be shown how a form of symmetry could arise from the learning procedure itself (for example from autoencoder-like unsupervised learning) or if a different formulation could eliminate the symmetry requirement. Encouraging cues come from the observation that denoizing autoencoders without tied weights often end up learning symmetric weights (Vincent et al., 2010). Another encouraging piece of evidence, also linked to autoencoders, is the theoretical result from Arora et al. (2015), showing that the symmetric solution minimizes the autoencoder reconstruction error between two successive layers of rectifying (ReLU) units, suggesting that symmetry may arise as the result of an additional objective function making successive layers form an autoencoder. Also, Lillicrap et al. (2014) show that the backpropagation algorithm for feedforward nets also works when the feedback weights are random, and that in this case the feedforward weight tend to “align” with the feedback weights.

Another practical issue is that we would like to reduce the negative impact of a lengthy relaxation to a fixed point, especially in the free phase. A possibility is explored by Bengio et al. (2016) and was initially discussed by Salakhutdinov and Hinton (2009) in the context of a stack of RBMs: by making each layer a good autoencoder, it is possible to make this iterative inference converge quickly after an initial feedforward phase, because the feedback paths “agree” with the states already computed in the feedforward phase.

Regarding synaptic plasticity, the proposed update formula can be contrasted with theoretical synaptic learning rules which are based on the Hebbian product of pre- and post-synaptic activity, such as the BCM rule (Bienenstock et al., 1982; Intrator and Cooper, 1992). The update proposed here is particular in that it involves the temporal derivative of the post-synaptic activity, rather than the actual level of postsynaptic activity.

Whereas our work focuses on a rate model of neurons, see Feldman (2012) for an overview of synaptic plasticity that goes beyond spike timing and firing rate, including synaptic cooperativity (nearby synapses on the same dendritic subtree) and depolarization (due to multiple consecutive pairings or spatial integration across nearby locations on the dendrite, as well as the effect of the synapse's distance to the soma). In addition, it would be interesting to study update rules which depend on the statistics of triplets or quadruplets of spikes timings, as in Froemke and Dan (2002) and Gjorgjievaa et al. (2011). These effects are not considered here but future work should consider them.

Another question is that of time-varying input. Although this work makes back-propagation more plausible for the case of a static input, the brain is a recurrent network with time-varying inputs, and back-propagation through time seems even less plausible than static back-propagation. An encouraging direction is that proposed by Ollivier et al. (2015) and Tallec and Ollivier (2017), which shows that computationally efficient estimators of the gradient can be obtained using a forward method (online estimation of the gradient), which avoids the need to store all past states in training sequences, at the price of a noisy estimator of the gradient.

## Author contributions

BS: main contributor to the theory developed in Section 3 and the experimental part (Section 5). YB: main contributor to the theory developed in Section 2.

### Conflict of interest statement

The authors declare that the research was conducted in the absence of any commercial or financial relationships that could be construed as a potential conflict of interest. The reviewer SF and handling Editor declared their shared affiliation, and the handling Editor states that the process nevertheless met the standards of a fair and objective review.

## Footnotes

^1^https://github.com/bscellier/Towards-a-Biologically-Plausible-Backprop

^2^For reasons of convenience, we use the same symbol *u* to mean both the set of units and the value of those units.

^3^The proof presented in Appendix A will show that *E*, *C*, and *F* need not satisfy Equation (20) but only Equation (26).

^4^Here, we are not considering numerical stability issues due to the encoding of real numbers with finite precision.

^5^Reccurent Back-propagation corresponds to Back-propagation Through Time (BPTT) when the network converges and remains at the fixed point for a large number of time steps.

^6^Recall that in our notations, the state variable *s* does not include the clamped inputs *x*, whereas *u* includes *x*.
