# Supplementary material for: Equilibrium Propagation: Bridging the Gap between Energy-Based Models and Backpropagation
Source: Front Comput Neurosci. 2017 May 4;11:24. doi: 10.3389/fncom.2017.00024 (PMC5415673; doi:10.3389/fncom.2017.00024)
Supplement: Supplementary file 1 [file DataSheet1.pdf]

# Appendix

## A Proof of the Gradient Formula (Theorem 1)

Here we prove Theorem 1 by directly computing the gradient of $J$. Another proof based on constrained optimization is proposed in Appendix B.

We first state and prove a lemma for a twice differentiable function $F(\theta, \beta, s)$. We assume that the conditions of the implicit function theorem are satisfied so that the fixed point $s_\theta^\beta$ is a continuously differentiable function of $(\theta, \beta)$. Since v does not play any role in the lemma, its dependence is omitted in the notations.

**Lemma 3** (Deterministic version)**.** *Let $F(\theta, \beta, s)$ be a twice differentiable function and $s_\theta^\beta$ a fixed point characterized by*

$$\frac{\partial F}{\partial s}\left(\theta, \beta, s_\theta^\beta\right) = 0. \tag{44}$$

*Then we have*

$$\left(\frac{d}{d\theta}\frac{\partial F}{\partial \beta}\left(\theta, \beta, s_\theta^\beta\right)\right)^T = \frac{d}{d\beta}\frac{\partial F}{\partial \theta}\left(\theta, \beta, s_\theta^\beta\right). \tag{45}$$

*The notations $\frac{\partial F}{\partial \theta}$ and $\frac{\partial F}{\partial \beta}$ are used to mean the partial derivatives with respect to the first and second arguments of $F$ respectively, whereas $\frac{d}{d\theta}$ and $\frac{d}{d\beta}$ represent the total derivatives with respect to $\theta$ and $\beta$ respectively (which include the differentiation path through $s_\theta^\beta$). The total derivative $\frac{d}{d\theta}$ (resp. $\frac{d}{d\beta}$) is performed for fixed $\beta$ (resp. fixed $\theta$).*

Interestingly, the variables $\theta$ and $\beta$ play symmetric roles in Eq. 45.

*A Concise Proof of Lemma 3.* Consider the function

$$G(\theta, \beta) := F\left(\theta, \beta, s_\theta^\beta\right), \tag{46}$$

which is the value of the total energy at the fixed point. The cross-derivatives of $G$ are transpose of each other:

$$\left(\frac{\partial^2 G}{\partial\theta\partial\beta}(\theta, \beta)\right)^T = \frac{\partial^2 G}{\partial\beta\partial\theta}(\theta, \beta). \tag{47}$$

This can be rewritten in the form

$$\left(\frac{d}{d\theta}\frac{d}{d\beta}F\left(\theta, \beta, s_\theta^\beta\right)\right)^T = \frac{d}{d\beta}\frac{d}{d\theta}F\left(\theta, \beta, s_\theta^\beta\right). \tag{48}$$

By the chain rule of differentiation we have

$$\frac{d}{d\beta}F\left(\theta, \beta, s_\theta^\beta\right) = \frac{\partial F}{\partial \beta}\left(\theta, \beta, s_\theta^\beta\right) + \frac{\partial F}{\partial s}\left(\theta, \beta, s_\theta^\beta\right) \cdot \frac{\partial s_\theta^\beta}{\partial \beta} = \frac{\partial F}{\partial \beta}\left(\theta, \beta, s_\theta^\beta\right). \tag{49}$$

Here we have used the fixed point condition (Eq. 44). Similarly we have

$$\frac{d}{d\theta}F\left(\theta, \beta, s_\theta^\beta\right) = \frac{\partial F}{\partial \theta}\left(\theta, \beta, s_\theta^\beta\right). \tag{50}$$

Plugging Eq. 49 and Eq. 50 in Eq. 48, we get

$$\left(\frac{d}{d\theta}\frac{\partial F}{\partial \beta}\left(\theta, \beta, s_\theta^\beta\right)\right)^T = \frac{d}{d\beta}\frac{\partial F}{\partial \theta}\left(\theta, \beta, s_\theta^\beta\right). \tag{51}$$

To provide the reader with more details and more insights, we propose another proof of Lemma 3 in which we explicitly compute the cross-derivatives of the function $G$ (defined in Eq. 46).

*Another Proof of Lemma 3.* First we differentiate the fixed point equation Eq. 44 with respect to $\beta$:

$$\frac{d}{d\beta}\,(44) \qquad \Rightarrow \qquad \frac{\partial^2 F}{\partial s \partial \beta}(\theta, \beta, s_\theta^\beta) + \frac{\partial^2 F}{\partial s^2}(\theta, \beta, s_\theta^\beta) \cdot \frac{\partial s_\theta^\beta}{\partial \beta} = 0. \tag{52}$$

Using again the chain rule of differentiation and Eq. 52, the transpose of the left-hand side of Eq. 45 can be rewritten

$$\frac{d}{d\theta}\frac{\partial F}{\partial \beta}\left(\theta, \beta, s_\theta^\beta\right) = \frac{\partial^2 F}{\partial \theta \partial \beta}\left(\theta, \beta, s_\theta^\beta\right) + \left(\frac{\partial s_\theta^\beta}{\partial \theta}\right)^T \cdot \frac{\partial^2 F}{\partial s \partial \beta}(\theta, \beta, s_\theta^\beta) \tag{53}$$

$$= \frac{\partial^2 F}{\partial \theta \partial \beta}\left(\theta, \beta, s_\theta^\beta\right) - \left(\frac{\partial s_\theta^\beta}{\partial \theta}\right)^T \cdot \frac{\partial^2 F}{\partial s^2}(\theta, \beta, s_\theta^\beta) \cdot \frac{\partial s_\theta^\beta}{\partial \beta}. \tag{54}$$

Similarly we differentiate the fixed point equation Eq. 44 with respect to $\theta$:

$$\frac{d}{d\theta}\,(44) \qquad \Rightarrow \qquad \frac{\partial^2 F}{\partial s \partial \theta}(\theta, \beta, s_\theta^\beta) + \frac{\partial^2 F}{\partial s^2}(\theta, \beta, s_\theta^\beta) \cdot \frac{\partial s_\theta^\beta}{\partial \theta} = 0. \tag{55}$$

and obtain the following form for the right-hand side of Eq. 45:

$$\frac{d}{d\beta}\frac{\partial F}{\partial \theta}(\theta, \beta, s_\theta^\beta) = \frac{\partial^2 F}{\partial \beta \partial \theta}(\theta, \beta, s_\theta^\beta) + \left(\frac{\partial s_\theta^\beta}{\partial \beta}\right)^T \cdot \frac{\partial^2 F}{\partial s \partial \theta}(\theta, \beta, s_\theta^\beta) \tag{56}$$

$$= \frac{\partial^2 F}{\partial \beta \partial \theta}(\theta, \beta, s_\theta^\beta) - \left(\frac{\partial s_\theta^\beta}{\partial \beta}\right)^T \cdot \frac{\partial^2 F}{\partial s^2}(\theta, \beta, s_\theta^\beta) \cdot \frac{\partial s_\theta^\beta}{\partial \theta}. \tag{57}$$

Clearly Eq. 54 is the transpose of Eq. 57. Hence the result. □

We have just proved that

$$\frac{d}{d\theta}\frac{\partial F}{\partial \beta}\left(\theta, \beta, s_\theta^\beta\right) = \frac{\partial^2 F}{\partial \theta \partial \beta}\left(\theta, \beta, s_\theta^\beta\right) - \left(\frac{\partial s_\theta^\beta}{\partial \theta}\right)^T \cdot \frac{\partial^2 F}{\partial s^2}\left(\theta, \beta, s_\theta^\beta\right) \cdot \frac{\partial s_\theta^\beta}{\partial \beta}. \tag{58}$$

It is worth mentioning that we can show with similar arguments that

$$\frac{d}{d\theta}\frac{\partial F}{\partial \beta}\left(\theta, \beta, s_\theta^\beta\right) = \frac{\partial^2 F}{\partial \theta \partial \beta}\left(\theta, \beta, s_\theta^\beta\right) - \frac{\partial^2 F}{\partial \theta \partial s}\left(\theta, \beta, s_\theta^\beta\right) \cdot \left(\frac{\partial^2 F}{\partial s^2}\left(\theta, \beta, s_\theta^\beta\right)\right)^{-1} \cdot \frac{\partial^2 F}{\partial s \partial \beta}\left(\theta, \beta, s_\theta^\beta\right). \tag{59}$$

Now we show that the gradient formula (Theorem 1) naturally arises from Lemma 3.

*Proof of Theorem 1.* According to Lemma 3 we have

$$\left(\frac{d}{d\theta}\frac{\partial F}{\partial \beta}\left(\theta, \mathrm{v}, \beta, s_{\theta,\mathrm{v}}^\beta\right)\right)^T = \frac{d}{d\beta}\frac{\partial F}{\partial \theta}\left(\theta, \mathrm{v}, \beta, s_{\theta,\mathrm{v}}^\beta\right). \tag{60}$$

Recall that the objective function is defined as

$$J(\theta, \mathrm{v}) = C\left(\theta, \mathrm{v}, s_{\theta,\mathrm{v}}^0\right) = \frac{\partial F}{\partial \beta}\left(\theta, \mathrm{v}, 0, s_{\theta,\mathrm{v}}^0\right). \tag{61}$$

Thus, for $\beta = 0$, the left-hand side of Eq. 60 represents the gradient of the objective function with respect to $\theta$:

$$\frac{\partial J}{\partial \theta}(\theta, \mathrm{v}). \tag{62}$$

On the other hand, the right-hand side of Eq. 60 represents the derivative of the function

$$\beta \mapsto \frac{\partial F}{\partial \theta}\left(\theta, \mathrm{v}, \beta, s_{\theta,\mathrm{v}}^{\beta}\right), \tag{63}$$

which, for $\beta = 0$, can be rewritten

$$\lim_{\beta \to 0} \frac{1}{\beta}\left(\frac{\partial F}{\partial \theta}\left(\theta, \mathrm{v}, \beta, s_{\theta,\mathrm{v}}^{\beta}\right) - \frac{\partial F}{\partial \theta}\left(\theta, \mathrm{v}, 0, s_{\theta,\mathrm{v}}^{0}\right)\right). \tag{64}$$

Therefore, combining Eq.62 and Eq. 64 we get the desired result. □

Finally, we prove Proposition 2.

*Proof of Proposition 2.* As in Lemma 3, we omit to write the dependence on v in the notations. Multiplying both sides of Eq. 52 on the left by $\left(\frac{\partial s_\theta^\beta}{\partial \beta}\right)^T$, we get

$$\left(\frac{\partial s_\theta^\beta}{\partial \beta}\right)^T \cdot \frac{\partial^2 F}{\partial s \partial \beta}(\theta, \beta, s_\theta^\beta) = -\left(\frac{\partial s_\theta^\beta}{\partial \beta}\right)^T \cdot \frac{\partial^2 F}{\partial s^2}(\theta, \beta, s_\theta^\beta) \cdot \frac{\partial s_\theta^\beta}{\partial \beta} \leq 0. \tag{65}$$

This inequality holds because $\frac{\partial^2 F}{\partial s^2}\left(\theta, \beta, s_\theta^\beta\right)$ is positive definite as $s_\theta^\beta$ is a local minimum of $F$. In particular, for $\beta = 0$, we get

$$\left(\left.\frac{\partial s_\theta^\beta}{\partial \beta}\right|_{\beta=0}\right)^T \cdot \frac{\partial C}{\partial s}\left(\theta, s_\theta^0\right) \leq 0. \tag{66}$$

Here we have used the fact that $C\left(\theta, s\right) = \frac{\partial F}{\partial \beta}\left(\theta, 0, s\right)$ when the value of $\beta$ is set to 0. Using the chain rule, we see that the left-hand side of Eq. 66 is the derivative of

$$\beta \mapsto C\left(\theta, s_\theta^\beta\right) \tag{67}$$

at the point $\beta = 0$. Hence the result. □

# B Reformulation of the Training Objective as a Constrained Optimization Problem

Here we give another proof for the gradient formula (Theorem 1). Considering v as fixed, and regarding $\theta$ and $s$ as the free parameters, we can frame the training objective (for a single training example v) as the following constrained optimization problem:

$$\text{find} \quad \arg\min_{\theta, s} C(\theta, \mathrm{v}, s) \tag{68}$$

$$\text{subject to} \quad \frac{\partial E}{\partial s}(\theta, \mathrm{v}, s) = 0. \tag{69}$$

Note that in more conventional machine learning algorithms, one only optimizes $\theta$, since the prediction is an *explicit* function of $\theta$. Here on the contrary, in the context of constrained optimization, the state $s$ is regarded as belonging to the set of free parameters that we optimize because the prediction is an *implicit* function of $\theta$ through the constraint Eq. 69.

As usual for constrained optimization problems, we introduce the Lagrangian

$$L(\theta, s, \lambda) := C(\theta, \mathrm{v}, s) + \lambda \cdot \frac{\partial E}{\partial s}(\theta, \mathrm{v}, s) \tag{70}$$

where $\lambda$ is the vector of Lagrange multipliers. We have omitted the explicit dependence on the data point v in the notation $L(\theta, s, \lambda)$, since this variable is considered fixed. Starting from the current parameter $\theta$, we first find $s^*$ and $\lambda^*$ such that

$$\frac{\partial L}{\partial \lambda}\left(\theta, s^*, \lambda^*\right) = 0 \tag{71}$$

and

$$\frac{\partial L}{\partial s}\left(\theta, s^*, \lambda^*\right) = 0, \tag{72}$$

and then we do one step of gradient descent on $L$ with respect to $\theta$, that is

$$\Delta\theta \propto -\frac{\partial L}{\partial \theta}\left(\theta, s^*, \lambda^*\right). \tag{73}$$

The first condition (Eq. 71) gives

$$\frac{\partial E}{\partial s}\left(\theta, \mathrm{v}, s^*\right) = 0 \quad \Rightarrow \quad s^* = s^0_{\theta,\mathrm{v}}. \tag{74}$$

Thus $s^*$ is the free fixed point. Injecting this into the second condition (Eq. 72) we get

$$\frac{\partial C}{\partial s}\left(\theta, \mathrm{v}, s^0_{\theta,\mathrm{v}}\right) + \lambda^* \cdot \frac{\partial^2 E}{\partial s^2}\left(\theta, \mathrm{v}, s^0_{\theta,\mathrm{v}}\right) = 0. \tag{75}$$

To solve this equation for $\lambda^*$, we introduce the total energy $F$ and use the definition of $C$ and $E$ in terms of $F$ (Eq. 26). We get

$$\frac{\partial^2 F}{\partial\beta\partial s}\left(\theta, \mathrm{v}, 0, s^0_{\theta,\mathrm{v}}\right) + \lambda^* \cdot \frac{\partial^2 F}{\partial s^2}\left(\theta, \mathrm{v}, 0, s^0_{\theta,\mathrm{v}}\right) = 0. \tag{76}$$

Comparing Eq. 76 and the transpose of Eq. 52 (evaluated at the point $\beta = 0$), and using the fact that $\frac{\partial^2 F}{\partial s^2}\left(\theta, \mathrm{v}, 0, s^0_{\theta,\mathrm{v}}\right)$ is invertible (it is positive definite since $s^0_{\theta,\mathrm{v}}$ is a local minimum of $s \mapsto F(\theta, \mathrm{v}, 0, s)$), we conclude that

$$\lambda^* = \left( \left.\frac{\partial s^\beta_\theta}{\partial \beta}\right|_{\beta=0} \right)^T, \tag{77}$$

which is the derivative of the fixed point with respect to $\beta$. Finally, using the values of $s^*$ and $\lambda^*$, and rewritting the Lagrangian (Eq. 70) in the form

$$L(\theta, s, \lambda) = \frac{\partial F}{\partial \beta}(\theta, \mathrm{v}, 0, s) + \lambda \cdot \frac{\partial F}{\partial s}(\theta, \mathrm{v}, 0, s), \tag{78}$$

we can compute the gradient of the Lagrangian:

$$\frac{\partial L}{\partial \theta}(\theta, s^*, \lambda^*) = \frac{\partial^2 F}{\partial\beta\partial\theta}(\theta, \mathrm{v}, 0, s^*) + \lambda^* \cdot \frac{\partial^2 F}{\partial s\partial\theta}(\theta, \mathrm{v}, 0, s^*) \tag{79}$$

$$= \frac{\partial^2 F}{\partial\beta\partial\theta}(\theta, \mathrm{v}, 0, s^0_{\theta,\mathrm{v}}) + \left( \left.\frac{\partial s^\beta_\theta}{\partial \beta}\right|_{\beta=0} \right)^T \cdot \frac{\partial^2 F}{\partial s\partial\theta}(\theta, \mathrm{v}, 0, s^0_{\theta,\mathrm{v}}) \tag{80}$$

$$= \left.\frac{d}{d\beta}\right|_{\beta=0} \frac{\partial F}{\partial \theta}(\theta, \mathrm{v}, \beta, s^\beta_{\theta,\mathrm{v}}). \tag{81}$$

Therefore Eq. 73 can be rewritten

$$\Delta\theta \propto -\lim_{\beta\to 0} \frac{1}{\beta}\left( \frac{\partial F}{\partial\theta}\left(\theta, \mathrm{v}, \beta, s^\beta_{\theta,\mathrm{v}}\right) - \frac{\partial F}{\partial\theta}\left(\theta, \mathrm{v}, 0, s^0_{\theta,\mathrm{v}}\right)\right). \tag{82}$$

# C Stochastic Framework

In this section we present a stochastic framework that naturally extends the deterministic framework studied in the paper. The analysis presented here could be the basis for a machine learning framework for spiking networks (Mesnard *et al.*, 2016).

Rather than the deterministic dynamical system Eq. 4, a more likely dynamics would include some form of noise. As suggested by Bengio and Fischer (2015), injecting Gaussian noise in the gradient system Eq. 4 leads to a Langevin dynamics, which we write as the following stochastic differential equation:

$$ds = -\frac{\partial F}{\partial s}(\theta, \mathrm{v}, \beta, s)dt + \sigma dB(t), \tag{83}$$

where $B(t)$ is a standard Brownian motion of dimension $\dim(s)$. In addition to the force $-\frac{\partial F}{\partial s}(\theta, \mathrm{v}, \beta, s)dt$, the Brownian term $\sigma dB(t)$ models some form of noise in the network. For fixed $\theta$, v and $\beta$, the Langevin dynamics Eq. 83 is known to converge to the Boltzmann distribution with temperature $T = \frac{1}{2}\sigma^2$ (consequence of the Fokker-Planck equation, a.k.a. Kolmogorov forward equation). For simplicity, here we assume that $\sigma = \sqrt{2}$, so that $T = 1$.

Let us denote by $p_{\theta,\mathrm{v}}^{\beta}$ the Boltzmann distribution corresponding to the energy function $F$. It is defined by

$$p_{\theta,\mathrm{v}}^{\beta}(s) := \frac{e^{-F(\theta,\mathrm{v},\beta,s)}}{Z_{\theta,\mathrm{v}}^{\beta}}, \tag{84}$$

where $Z_{\theta,\mathrm{v}}^{\beta}$ is the partition function

$$Z_{\theta,\mathrm{v}}^{\beta}(s) := \int e^{-F(\theta,\mathrm{v},\beta,s)} ds. \tag{85}$$

After running the dynamics Eq. 83 for long enough, we can obtain a sample $s^0$ from the stationary distribution $p_{\theta,\mathrm{v}}^{0}$ in the free phase (with $\beta = 0$) and similarly we can obtain a sample $s^\beta$ from $p_{\theta,\mathrm{v}}^{\beta}$ in the weakly-clamped phase (with $\beta > 0$). Theorem 4 below will show that $\frac{1}{\beta}\left(\frac{\partial F}{\partial \theta}\left(\theta, \mathrm{v}, \beta, s^\beta\right) - \frac{\partial F}{\partial \theta}\left(\theta, \mathrm{v}, 0, s^0\right)\right)$ is an unbiased estimator of the gradient of the following objective function:

$$\widetilde{J}(\theta, \mathrm{v}) := \mathbb{E}_{\theta,\mathrm{v}}^{0}\left[C(\theta, \mathrm{v}, s)\right]. \tag{86}$$

Here $\mathbb{E}_{\theta,\mathrm{v}}^{\beta}$ denotes the expectation over $s \sim p_{\theta,\mathrm{v}}^{\beta}(s)$ and $C$ is the cost function.

**Theorem 4** (Stochastic version)**.**

$$\frac{\partial \widetilde{J}}{\partial \theta}(\theta, \mathrm{v}) = \lim_{\beta \to 0} \frac{1}{\beta}\left(\mathbb{E}_{\theta,\mathrm{v}}^{\beta}\left[\frac{\partial F}{\partial \theta}(\theta, \mathrm{v}, \beta, s)\right] - \mathbb{E}_{\theta,\mathrm{v}}^{0}\left[\frac{\partial F}{\partial \theta}(\theta, \mathrm{v}, 0, s)\right]\right), \tag{87}$$

Theorem 4 generalizes Theorem 1 to the stochastic framework and is a consequence of Lemma 5 below (which itself is a generalization of Lemma 3).

**Lemma 5** (Stochastic version)**.** *Let $F(\theta, \beta, s)$ be a twice differentiable function and $p_\theta^\beta$ the Boltzmann distribution defined by*

$$p_{\theta}^{\beta}(s) := \frac{e^{-F(\theta,\beta,s)}}{Z_{\theta}^{\beta}}, \tag{88}$$

*where $Z_\theta^\beta$ is the partition function*

$$Z_{\theta}^{\beta}(s) := \int e^{-F(\theta,\beta,s)}, \tag{89}$$

*and $\mathbb{E}_\theta^\beta$ the expectation over $s \sim p_\theta^\beta(s)$. Then we have*

$$\left(\frac{d}{d\theta}\mathbb{E}_{\theta}^{\beta}\left[\frac{\partial F}{\partial \beta}(\theta, \beta, s)\right]\right)^T = \frac{d}{d\beta}\mathbb{E}_{\theta}^{\beta}\left[\frac{\partial F}{\partial \theta}(\theta, \beta, s)\right]. \tag{90}$$

*As in Theorem 3, the variables $\theta$ and $\beta$ play symmetric roles in Eq. 90.*

*Proof of Lemma 5.* The differentials of the log partition function are equal to

$$\frac{d}{d\beta}\ln\left(Z_\theta^\beta\right) = -\mathbb{E}_\theta^\beta\left[\frac{\partial F}{\partial \beta}(\theta,\beta,s)\right] \tag{91}$$

and

$$\frac{d}{d\theta}\ln\left(Z_\theta^\beta\right) = -\mathbb{E}_\theta^\beta\left[\frac{\partial F}{\partial \theta}(\theta,\beta,s)\right]. \tag{92}$$

Therefore

$$\left(\frac{d}{d\theta}\mathbb{E}_\theta^\beta\left[\frac{\partial F}{\partial \beta}(\theta,\beta,s)\right]\right)^T = -\left(\frac{d}{d\theta}\frac{d}{d\beta}\ln\left(Z_\theta^\beta\right)\right)^T = -\frac{d}{d\beta}\frac{d}{d\theta}\ln\left(Z_\theta^\beta\right) = \frac{d}{d\beta}\mathbb{E}_\theta^\beta\left[\frac{\partial F}{\partial \theta}(\theta,\beta,s)\right]. \tag{93}$$

□

Finally we state a result similar to Proposition 2 in the stochastic framework, which shows that for a small $\beta > 0$ the 'nudged' distribution $p_{\theta,\mathrm{v}}^\beta$ is better than the 'free' distribution $p_{\theta,\mathrm{v}}^0$ in terms of expected cost.

**Proposition 6** (Stochastic version)**.** *The derivative of the function*

$$\beta \mapsto \mathbb{E}_{\theta,\mathrm{v}}^\beta\left[C(\theta,\mathrm{v},s)\right] \tag{94}$$

*at the point $\beta = 0$ is non-positive.*

*Proof.* The derivative of Eq. 94 is

$$-\mathbb{E}_{\theta,\mathrm{v}}^0\left[(C(\theta,\mathrm{v},s))^2\right] + \left(\mathbb{E}_{\theta,\mathrm{v}}^0\left[C(\theta,\mathrm{v},s)\right]\right)^2 = -\mathrm{Var}_{\theta,\mathrm{v}}^0\left[C(\theta,\mathrm{v},s)\right] \le 0, \tag{95}$$

where $\mathrm{Var}_{\theta,\mathrm{v}}^0$ represents the variance over $s \sim p_{\theta,\mathrm{v}}^0(s)$. □
